# Impacted Third Molar in a Mummy

**Published:** 1901-05

**Authors:** 


Impacted Third Molar in a Mummy.—Mr. William Hern showed to the Society and presented to the Museum some specimens of mummy jaw-bones (two mandibles and two superior maxillary bones) brought from Egypt by Mr. Blackden (to whom the Society is indebted for one of the mummy heads in its possession), who states that the bones are of extreme age, having been taken from tombs at Beni Hassan, where the doubled-up burials of the earliest inhabitants of Egypt are found.

One specimen, the mandible of a young adult, shows a well-marked impacted wisdom-tooth on the right side; this tooth is erupted in a semi-recumbent position, with its anterior cusps impinging against the distal surface of the second lower molar. The posterior surface of the crown is exposed to mastication, and a small, well-marked facet on the distal surface of the tooth shows that it has been in antagonism during mastication with the upper wisdom-tooth.

The teeth generally are sound, and about normal in size, although some, and especially the sixth-year molars, are much worn by surface attrition. On the lingual surfaces of the molars some thin ledges of salivary calculus are still attached. There is no sign of serious inflammation having occurred about the alveolar margin surrounding the wisdom tooth.—*Transactions Odontological Society of Great Britain.*

This interesting specimen of a human jaw, the age of which is estimated to be at least four thousand years, demonstrates very conclusively that at that early period in the world's history such a thing as an impacted molar was, probably, not uncommon. The position of the tooth, in the illustration given, is one quite familiar to all dentists of the present era.

The impaction of the third molar has been used as one of the arguments that the third molar is gradually undergoing a process of extinction as one of the regular series. While four thousand years may be a very short time in the history of the race and its evolution from one stage to another, it is a satisfaction to know that at that remote period the impacted third molar was not an unknown irregularity, and that it presented in form and position the peculiar characteristics of that tooth in the present age.—TRUMAN.

